# Asthma: Bowled Over by Dust

**Published:** 2006-03

**Authors:** Victoria McGovern

A common substance in house dust is a major risk factor for asthma, according to work reported in the 1 December 2005 issue of the *American Journal of Respiratory and Critical Care Medicine*. The study charted room-by-room distribution of endotoxin, a lipid-like material that comes from the surface of bacteria, in the first nationwide snapshot of exposure in American homes. Then researchers looked at the relationship between household endotoxin exposure and the presence of allergic symptoms (such as hay fever) and asthma.

The field sampling showed that endotoxin exposure “isn’t something that’s only limited to inner-city homes or to homes that are dirty or to homes in certain parts of the country,” says coauthor Darryl Zeldin, a senior investigator in the NIEHS Laboratory of Respiratory Biology. “Almost all the homes in the nation have detectable levels of endotoxin in multiple locations.”

The study used data from the U.S. National Survey of Lead and Allergens in Housing, which ran from July 1998 through August 1999. In this survey, field personnel collected dust samples (and later analyzed their composition), recorded demographic and health data, and conducted visual inspections of a nationwide representative sample of 831 dwellings. Zeldin and colleagues were then able to analyze the health impacts of individual dust components from sources including not only bacteria, but also dogs, cats, rodents, cockroaches, dust mites, and fungi.

Although endotoxin was found in all of the homes studied, concentrations varied considerably from home to home and from room to room within a given home. Concentrations were on average highest on kitchen floors, but it was the bedroom exposure that was most highly associated with residents having doctor-diagnosed asthma, experiencing asthma symptoms such as wheezing, or using asthma medications. Zeldin says, “Endotoxin exists on [dust] particles that can go airborne with activity—vacuuming, sleeping on their bed or with their face in the bedding, kids playing on their beds or on the floor.” The asthma–endotoxin link persisted at all exposure levels, with symptoms more likely with higher exposure.

The relationship of asthma to endotoxin was strong for adults in the study, but not for children. However, the authors say the original survey was not designed to collect information on when or how long people had been exposed, nor did it have sufficient statistical power to examine these relationships in young children.

So far, other work in children has mostly led to more questions. The answers may lie in inflammation of the airways rather than activation of allergy’s histamine responses. “What is well understood is that endotoxin exposure worsens airway inflammation and symptoms in people with asthma,” says Andy Liu, an asthma researcher at the National Jewish Medical and Research Center in Denver. “This investigation . . . supports endotoxin’s relationship with asthma as a toxic one, and is consistent with what has been reported with infant wheezing. What is perplexing is that, in older children, the association of allergy-associated asthma with endotoxin becomes a negative one: more exposure, less disease.”

That kind of observation forms the root of the hygiene hypothesis—that exposure to infections in early life helps build an immune system that is less prone to allergic diseases in later life. But does an exposure–allergy relationship tie in with asthma?

“The question with the hygiene hypothesis is, can we extend it from ‘infection’ to endotoxin—which comes from bacteria—and can we extend it from allergy to asthma?” says lead author Peter Thorne, director of the Environmental Health Sciences Research Center at the University of Iowa. The current study suggests not, he says: “What we found is that endotoxin is causing symptoms of asthma in both those who are allergic and those who are not. So endotoxin exposure is not protecting people from asthma.”

## Figures and Tables

**Figure:**
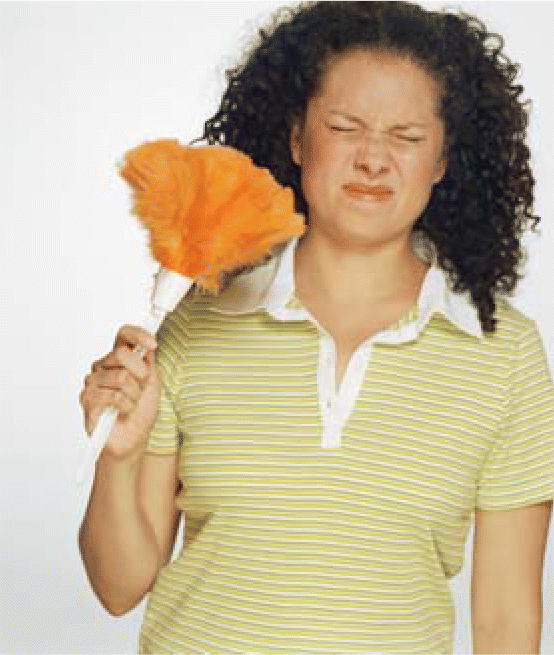
Nothing to sneeze at. Endotoxin, commonly found in household dust, is a ubiquitous risk factor for asthma.

